# The Immune-Stimulating and Anti-Diabetic Effects of *Allium hookeri* Leaves Grown in a Plant Factory with Artificial Lights in Immunosuppressed Obese C57BL/6 Mice

**DOI:** 10.3390/ph17010091

**Published:** 2024-01-09

**Authors:** Jieun Jung, Ji-Su Kim, Un-Yul Jeong, Ui-Jin Bae, Mina Kim, Shin-Young Park, In-Guk Hwang, Jeong-Wook Heo, Chang-Ki Shim, Jun-Sang Ham, Sung-Hyen Lee

**Affiliations:** 1 Department of Agro-Food Resources, National Institute of Agricultural Sciences, Rural Development Administration, Wanju 55365, Republic of Korea; jjempm@korea.kr (J.J.); ver0218@korea.kr (J.-S.K.); juw325@naver.com (U.-Y.J.); e-jin86@hanmail.net (U.-J.B.); lucidminakim@gmail.com (M.K.); soyoenj@korea.kr (S.-Y.P.); ighwang79@korea.kr (I.-G.H.); 2 Department of Agricultural Engineering, National Institute of Agricultural Sciences, Rural Development Administration, Wanju 55365, Republic of Korea; 3 Department of Agricultural Environment, National Institute of Agricultural Sciences, Rural Development Administration, Wanju 55365, Republic of Korea; ckshim@korea.kr; 4 Department of Animal Biotechnology and Environment, National Institute of Animal Science, Rural Development Administration, Wanju 55365, Republic of Korea; hamjs@korea.kr

**Keywords:** *Allium hookeri* leaf, artificial light, immune-enhancing, anti-diabetic effect

## Abstract

We investigated the immune-stimulating and anti-diabetic effects of *Allium hookeri* leaves grown in a plant factory with artificial lights. The immunomodulatory effects of *A. hookeri* leaves’ ethanol extracts were evaluated with immune-related hematological factors in blood, the proliferation of splenocytes, NK cell activity, IgG and cytokine levels, and their mechanisms in immunosuppressed obese mice. Anti-diabetic effects were determined by the inhibitory activity against α-amylase and α-glucosidase in vitro and fasting blood glucose levels and biochemical factors in the serum of immunosuppressed obese mice. *A. hookeri* leaf extracts increased WBC and LYM counts, the proliferation of splenocytes, and serum IgG and IL-1β concentrations compared to those of the NC group, which was used as a negative control. *A. hookeri* leaf extracts also improved serum HDL levels while they decreased the activities of digestive enzymes, fasting blood glucose, and biochemical factors (ALT, AST, T-Chol, TG, LDL, and GLU). The expressions of IL-1β, JNK, c-Jun, p65, and iNOS in the thymus of immunosuppressed mice were activated by the treatment of *A. hookeri* leaf extracts. The results suggest that *A. hookeri* leaves grown in a plant factory with artificial lights also have immune-stimulatory and anti-diabetic effects and can be used as novel functional supplements to control related diseases and to improve public health.

## 1. Introduction

Obesity is a global health issue. As per World Health Organization standards, a body mass index (BMI) of above 25 kg/m^2^ is classified as overweight, and a BMI exceeding 30 kg/m^2^ is classified as obese [1]. According to a recent report, the obesity rate in Korean adults was 37.2%, and men and women exhibited a rate of 44.8% and 29.5% in 2021, respectively [2]. Obesity is a harmful health condition and is a consequence of excess fat accumulation [1]. It increases the risk of developing other diseases, including type 2 diabetes, metabolic syndrome, dyslipidemia, atherosclerosis, hypertension, cardiovascular disease, and cancer [3,4]. Obesity induces insulin resistance and affects β-cell insulin secretory function. Accordingly, a high prevalence of obesity is accountable for the increment in the prevalence of type 2 diabetes [5]. Also, it produces impairments in the immune response, such as a higher rate of infections, a delay in wound healing, and a lower production of antibodies [6].

The regulation of immune response plays an essential role in preventing and treating disease [7]. Immunity is divided into innate and adaptive immunities. The innate immune system is the first line of immune protection and acts initially to recognize and eliminate pathogens [8]. The innate immunity produces chemokines, cytokines, such as tumor necrosis factor (TNF), interleukin-1 (IL-1), and IL-6, and natural killer (NK) cells [9]. The adaptive immune system has evolved to compensate for the limited recognition of the innate immune system [8]. The adaptive immune system includes T lymphocytes, which are the effectors of cellular immune responses, and B lymphocytes, which are antibody-producing cells [10].

*Allium hookeri* Thwaites, belonging to the family Liliaceae, like garlic, onion, scallion, leek, and chive, is distributed throughout Korea, Sri Lanka, India, Myanmar, and southwestern China [11,12]. *A. hookeri* has sweet, bitter, and spicy flavors and has been consumed as a food and flavoring ingredient, including fermented foods, spices, seasonings, salads, and soups [11,12,13]. *A. hookeri* has underground rhizomes that produce bright fibrous roots and evergreen, thick, and linear leaves [12]. It is reported that *A. hookeri* has diverse pharmacological attributes, including antioxidant [12], anti-inflammatory [12,14], antimicrobial [12], anti-diabetic [11,12], anti-obesity [11,12,15], bone formation-improving [12], anticancer [11], anti-asthmatic [11], and neuroprotective effects [12].

Recently, climate change has caused crop damage and problems with food stability. Several laboratories have researched plant factories to resolve this problem. Plant factories automatically control the essential elements of plant growth, such as temperature, humidity, and carbon dioxide, in indoor spaces [16]. Artificial light is an important factor in a plant factory. An artificial light contains a high-pressure sodium lamp, metal halide lamp, incandescent lamp, fluorescent lamp (FL), and light-emitting diode (LED) [17]. FL emits a wide spectrum of light of 400–700 nm and is commonly used in greenhouses [18]. However, FLs have low luminous efficiency, high power consumption, and incomplete spectral distribution [19]. For that reason, LEDs were introduced and are extensively used in plant factories. LEDs have a single-spectrum wavelength, extended life, lower power consumption, little heat production, and directional light emission [18,20]. Red (600–700 nm) and blue (400–500 nm) LEDs are used for the cultivation of spinach, lettuce, and radish and have a high absorbance in plant leaves [20]. The mixed red and blue LEDs promote plant growth [20]. Also, FLs and LEDs influence bioactive compounds and the condition of a plant. An orange or green LED increases phenolic compounds, α-carotene, and anthocyanin in baby leaf lettuce, and a blue, red, or green LED increases its total flavonoid content [21]. The light intensity and photoperiod changed the growth condition, the leaf size and shape, the total phenolic content, and the antioxidant level of the ‘Cheongchima’ lettuce [22].

Previous studies researched the in vitro functionality of *A. hookeri* leaves and roots grown in a hydroponic plant factory using FLs and LEDs. FLs and the mixed blue, red, and white LEDs improved the antioxidant, immune-stimulating, and anti-diabetic effects of *A. hookeri* leaves and roots in in vitro studies [23]. Diverse conditions of artificial lights such as FLs, red LEDs, blue LEDs, and mixed red and blue LEDs enhance the radical-scavenging activity, enzymatic antioxidant capacity, and anti-inflammatory activity of *A. hookeri* leaves in in vitro studies [24]. However, there is scant information available on the functionality of *A. hookeri* leaves grown in plant factories with artificial lights. Thus, we investigated the bioactive components and anti-diabetic and immune-enhancing effects of *A. hookeri* leaves grown in a plant factory using in vitro and in vivo experiments with a mouse model. Cycloalliin, which is a bioactive component in *A. hookeri* leaves, was assessed using LC/MS, and the inhibition of diastatic enzymes, including α-amylase and α-glucosidase, was measured in in vitro studies. Also, we used an immunosuppressed obese mouse model and investigated body and organ weights, fasting blood glucose, toxicological factors, lipid profile, splenocyte proliferation, immunoglobulin and cytokines in serum, and natural killer (NK) cell activity using the in vivo model.

## 2. Results and Discussion

### 2.1. Concentration of Alliin and Cycloalliin

**Figure 1:**
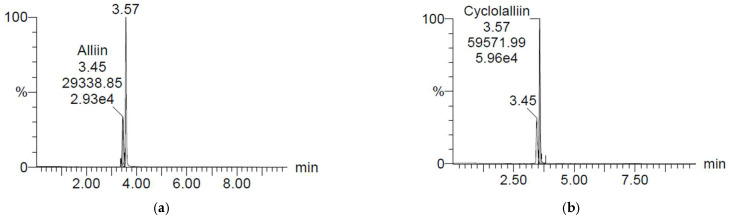
Chromatograms of (**a**) alliin and (**b**) cycloalliin standards at 1000 ppb (ng/mL) analyzed by LC/MS.

*Allium* sp. contains high flavonoids, phytosterols, and organosulphur compounds. It has been reported that organosulphur compounds have beneficial effects and bioactive functions [12]. Organosulphur compounds in *A. hookeri* include cycloalliin, alliin, isoalliin, alliicin, and methiin [12,25]. Alliin has anti-inflammatory and anti-helicobacter activities and promotes bone formation [12], and cycloalliin has antioxidant and hypolipidemic activities [25]. Alliin ((+)-S-ally-L-cysteine sulfoxide) is the first compound reported from *Allium* sp. as one of the major components of *A. hookeri* [12,15]. Cycloalliin ((3R,5S)-5-methyl-1,4-thiazane-3-carboxylic acid) as a main compound of *A. hookeri* is much more stable and flavorless compared to the other organosulphur compounds [12,26]. So, alliin and cycloalliin were analyzed as biological markers of *A. hookeri* leaves grown in OF and plant factories. Figure 1 reveals chromatograms of alliin and cycloalliin standards (C_6_H_11_NO_3_S) analyzed by the research team from Jeonbuk National University (Jeonju). Regression equations (y) of alliin and cycloalliin were 228.62x + 569.29 and 157.62x + 549.36, respectively, and their correlation coefficients (R2) were 1.

The concentration of alliin was about 8–23 times higher in *A. hookeri* leaves grown in the outfield (1669.66 μg/g sample) than in *A. hookeri* leaves grown in a plant factory (70.93–200.19 μg/g sample), as shown in Table 1. Similarly, the cycloalliin level was 3 times higher in the *A. hookeri* leaves grown in the outfield (442.20 μg/g sample) than in other *A. hookeri* leaves (65.17–143.77 μg/g sample), followed by the *A. hookeri* leaves grown in the plant factory with FL (Table 1).

### 2.2. Effects of A. hookeri Leaf Extracts on α-Amylase and α-Glucosidase Inhibitory Activities

**Figure 2:**
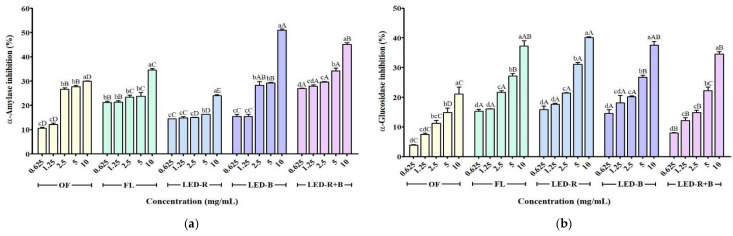
Anti-diabetic enzyme effect of *A. hookeri* leaves grown in the plant factory with artificial lights. (**a**) α-amylase inhibitory activity; (**b**) α-glucosidase inhibitory activity. OF, *A. hookeri* leaves grown in outfield; FL, *A. hookeri* leaves grown in plant factory with fluorescent lamp; LED-R, *A. hookeri* leaves grown in plant factory with LED-R; LED-B, *A. hookeri* leaves grown in plant factory with LED-B; LED-R+B, *A. hookeri* leaves grown in plant factory with LED-R+B. Data were expressed as the mean ± SEM. ^a–d^ Means with different letters within the sample are significantly different (*p* < 0.05). ^A–D^ Means with different letters at the same concentration of the extracts are significantly different (*p* < 0.05) among groups by Duncan’s multiple range test.

Diabetes can be prevented through the control of the glucose level in blood. α-Amylase and α-glucosidase are the key enzymes responsible for the hydrolysis of starch into simple sugars [27,28]. α-Amylase is secreted by the pancreas, and α-glucosidase is an intestinal cell membrane enzyme [27]. The inhibition of two enzymes can decrease carbohydrate digestion, reduce the rate of glucose absorption and food calorie intake, and delay fat accumulation [29]. The inhibitory effects of *A. hookeri* leaf extracts on α-amylase and α-glucosidase activity are shown in Figure 2.

Different concentrations (0.625, 1.25, 2.5, 5, and 10 mg/mL) of *A. hookeri* leaf extracts were tested for the inhibition of α-amylase activity. The α-amylase inhibitory activity of OF, FL, LED-R, LED-B, and LED-R+B were 10.6–30.0, 21.3–34.6, 14.5–24.1, 15.5–51.1, and 27.0–45.1%, respectively. Generally, LED-R+B showed the highest α-amylase inhibition among *A. hookeri* leaf extracts. The IC_50_ values of the α-amylase inhibitory activity in OF, FL, LED-R, LED-B, and LED-R+B were 49.92, 283.6, 2924, 11.74, and 31.24 mg/mL, respectively. Previous studies demonstrated that the IC_50_ values of α-amylase on *Hibiscus sabdariffa* extracted with water, methanol, and ethanol were 5.74, 3.88, and 3.86 mg/mL, and mulberry root bark 70% ethanol and hot water extracts were 7.86 and 20.10 mg/mL [28,29]. The IC_50_ values of the α-amylase inhibitory activity of *Kedrostis africana* aqueous and ethanolic extracts were 439.45 and 949.75 μg/mL [30]. The α-amylase inhibitory activities of methanol extracts of plants (*Commiphora wightii*, *Trachyspermum ammi*, *Nigella sativa*, *Coffea arabica*, *Linum usitatissimum*, *Cuminum cyminum*, and *Ruta graveolens*) were 54.5, 53.0, 13.0, 32.5, 10.0, 45.5, and 73.0% at 500 μg/mL [31]. Our study determined that the α-amylase inhibitory activity at 625 μg/mL of *A. hookeri* leaves grown in the outfield and a plant factory with FL, LED-R, -B, and -R+B was 10.6, 21.3, 14.5, 15.5, and 27.0%.

The α-glucosidase inhibitory activities of OF, FL, LED-R, LED-B, and LED-R+B at 0.625–10 mg/mL ranged from 3.9 to 21.1, 15.3 to 37.3, 15.9 to 40.2, 14.6 to 37.6, and 8.1 to 34.5%, respectively. The α-glucosidase inhibitory activity of LED-R showed the highest value at 10 mg/mL. Previous studies demonstrated that the IC_50_ values of α-glucosidase on *H. sabdariffa* extracted with water, methanol, and ethanol were 3.65, 1.59, and 1.85 mg/mL, respectively, and the values of mulberry root bark 70% ethanol and hot water extracts were 0.12 and 1.15 mg/mL, respectively [28,29]. The IC_50_ values of the α-glucosidase inhibitory activity in OF, FL, LED-R, LED-B, and LED-R+B were 90.26, 34.09, 23.65, 34.68, and 27.74 mg/mL, respectively. The IC_50_ value of the α-glucosidase inhibition activity of *Ipomoea reptans* leaf ethanolic extract was 0.26 mg/mL, and the value was 47.8 μg/mL in *Olax imbricata* Roxb. root methanol extract [32,33]. The IC_50_ values of the α-glucosidase inhibitory activity of *K. africana* aqueous and ethanolic extracts were 486.92 and 157.99 μg/mL [30]. The α-Glucosidase inhibitory activity of methanol extracts of plants (*C. wightii*, *T. ammi*, *N. sativa*, *C. arabica*, *L. usitatissimum*, *C. cyminum*, and *R. graveolens*) were 91.0, 16.0, 3.0, 14.0, 15.0, 35.0, and 71.0% at 500 μg/mL, respectively [31]. *A. hookeri* leaf extract contains organosulphur compounds as bioactive compounds. The inhibition against α-amylase and α-glucosidase can decrease starch digestion and the energy absorbed into the body [33], which can lead to weight loss and is useful in the treatment of obesity [30]. It may explain the high inhibitory activities against digestive enzymes in *A. hookeri* leaf extracts, which can be attributed to their anti-diabetic effect.

### 2.3. Effects of A. hookeri Leaf Extracts on Body and Organ Weights of the Immunosuppressed Obese Mice

Table 2 shows the body and tissue weights of both normal and immunosuppressed obese mice. The initial body weights of mice were not significant in all groups. However, final body weights were significantly lower in the NC group than in the NOR group due to CPA treatment, which induces immunosuppression and weight loss in spite of a high-fat diet for the immunosuppressed obese model [34]. The final body weights of mice treated with *A. hookeri* leaf extracts increased and were comparable to those of the NOR group, which was not immunosuppressed with CPA. In a previous study, the administration of *A. hookeri* leaf and root extracts improved the final body weights of immunosuppressed mice [35]. *A. hookeri* leaf groups showed higher spleen and thymus weights than those of the NC group among all immunosuppressed mice and reached those of the NOR group. A high-fat diet increased the liver, heart, epididymal fat, and pancreas weights of the mice compared to those of the NOR group, which was fed a normal diet. However, *A. hookeri* leaf extracts reduced the liver, heart, epididymal fat, and pancreas weights in obese mice, and a significant difference from the NC group was found in the heart and epididymal fat weights. Aqueous and ethanol extract (100 and 200 mg/kg BW) of *A. hookeri* leaves showed similar patterns by increasing spleen index and decreasing liver and epididymal fat indexes in type 2 diabetic mice [36]. *A. hookeri* root ethanolic extract also decreased liver and epididymal fat weights in high-fat-diet-induced obese mice [15]. A previous study reported that the administration of hydrolyzed and fermented *Platycodon grandiflorum* extract increased the spleen and thymus weights in CPA-induced immunosuppressed BALB/c mice [34], which are important immune organs playing immune homeostasis [37].

CPA causes weight loss in the immune organs, such as the spleen and thymus, which are used as biomarkers of immunosuppressed conditions [37,38]. Our data demonstrated that all *A. hookeri* leaves grown in the outfield and plant factory improved the decreased spleen and thymus indexes induced by CPA. Aqueous and ethanolic extracts of Welsh onion reduced the epididymal fat weight of high-fat-diet-fed mice [3], which is a component of visceral fat and is considered an indicator of obesity [39]. The results suggest that all *A. hookeri* leaves grown in both the outfield and the plant factory with artificial lights can prevent obesity by avoiding the accumulation of epididymal fat. The pancreas weights of *A. hookeri* leaf groups were lower than that of the NC obese group. It has been reported that pancreatic β-cell mass is 50% greater in people with obesity than in people who are lean, and weight gain in mice fed a high-fat diet is associated with a high proliferation in β-cell mass [5].

### 2.4. Effects of A. hookeri Leaf Extracts on Fasting Blood Glucose of the Immunosuppressed Obese Mice

**Figure 3:**
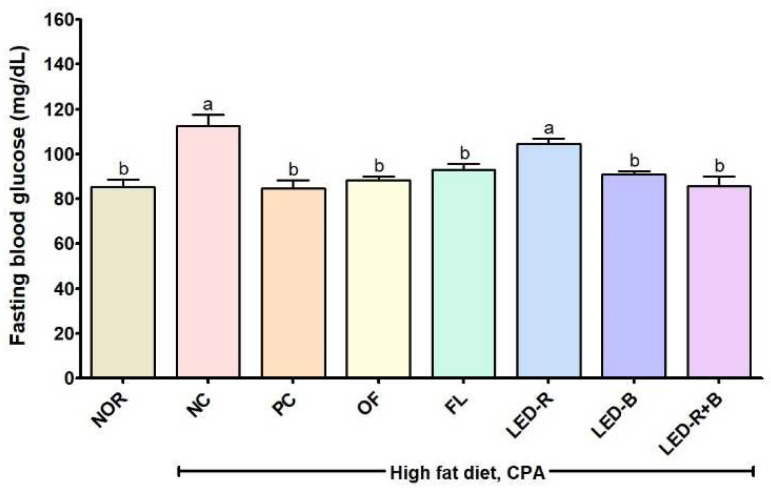
Effects of *A. hookeri* leaf extracts on fasting blood glucose levels in immunosuppressed obese mice. NOR, normal control fed normal diet; immunosuppressed obese groups treated with CPA and high-fat diet. NC, negative control; PC, positive control (β-glucan 50 mg/kg BW); OF, *A. hookeri* leaves grown in outfield (300 mg/kg BW); FL, *A. hookeri* leaves grown under fluorescent lamp (300 mg/kg BW); LED-R, *A. hookeri* leaves grown under LED-R (300 mg/kg BW); LED-B, *A. hookeri* leaves grown under LED-B (300 mg/kg BW); LED-R+B, *A. hookeri* leaves grown under LED-R+B (300 mg/kg BW). Data were expressed as the mean ± SEM. ^a,b^ Different letters are significantly different among groups at *p* < 0.05 by Duncan’s multiple range test.

Figure 3 shows the fasting blood glucose levels of the immunosuppressed obese mice treated with *A. hookeri* leaf extracts after 16 h starvation. The fasting blood glucose value was significantly higher in the NC group compared to the NOR group (112.5 vs. 85.4 mg/dL). However, the blood glucose levels significantly decreased in the mice treated with the *A. hookeri* leaf extracts except for the LED-R extract. Fasting blood glucose levels were lower in OF, FL, LED-B, and LED-R+B groups by 21.4, 17.3, 19.2, and 23.8%, respectively, compared with that of the NC group. In the previous studies, aqueous and ethanolic extracts of *A. hookeri* roots reduced the fasting blood glucose concentrations in type 2 C57BL/J-db/db mice [36], and onion powder decreased the fasting blood glucose level in streptozotocin-induced diabetic Wistar albino rats [40]. In this study, *A. hookeri* leaves cultured in the plant factory with various artificial lights affected fasting blood glucose levels in immunosuppressed obese mice and showed similar effects to the PC group on fasting blood glucose levels of high-fat-diet-induced obese C57BL/6 mice.

### 2.5. Effects of A. hookeri Leaf Extracts on Hematological Factors of the Immunosuppressed Obese Mice

Table 3 shows the hematological factors of CPA-induced immunosuppressed obese mice. They are used as biomarkers in the diagnosis of organ injuries and an aid in diagnosing infections [41]. Leukocytes participate in immune and inflammatory processes, and LYM is mostly a mediator of adaptive immunity [41]. The change in leukocyte counts may indicate infectious and/or inflammatory conditions [41]. RBC, HGB, HCT, MCV, PLT, WBC, LYM, and MONO levels decreased in the blood of CPA-induced immunosuppressed obese mice, while MCH, MCHC, NEU, EOS, and BASO levels increased in the NC group compared with the NOR group. The administration of *A. hookeri* leaf extracts improved the levels of impaired hematological factors. In previous studies, the administration of onion skin increased the concentration of MCV, PLT, WBC, LYM, and MONO values in CPA-induced immunosuppressed mice [42], and *Sargassum horneri* and *Phellinus baumii* recovered counts of RBC, WBC, and PLT and the concentration of HGB and LYM in the immunosuppression model [43,44]. Obesity produces impairments in the immune response, such as a higher rate of infection, delay of wound healing, and lower production of antibodies [6]. In the present study, *A. hookeri* leaf extracts also showed immune-enhancing and anti-obesity effects in immunosuppressed obese mice.

### 2.6. Effects of A. hookeri Leaf Extracts on Biochemical Factors of the Immunosuppressed Obese Mice

Table 4 shows the biochemical factors in CPA-induced immunosuppressed obese mice. These parameters provide important information on the clinical status, nutritional balance, and metabolic functioning of the organs [41]. In the NC group, CPA treatment increased ALT and AST levels and high-fat-diet-induced high T-Chol and LDL levels compared with the NOR group. However, *A. hookeri* leaf extracts reduced the levels of ALT, AST, T-Chol, TG, LDL, and GLU levels while increasing the HDL level compared with the NC group and recovered to the values of the NOR group. Significant differences between the NC and *A. hookeri* leaf groups were found in ALT, AST, and TG concentrations. Interestingly, the GLU level was significantly lower in the groups treated with *A. hookeri* leaves grown in the plant factory with artificial lights, and the HDL level was significantly higher in the OF, FL, and LED-B groups than in the high-fat-diet-induced obese mice (NC group). There was no significant difference found in the HbA1c levels of immunosuppressed obese mice. A previous study reported that *A. hookeri* leaves extracted with 80% ethanol reduced ALT, AST, T-Chol, TG, and LDL and raised HDL in the serum of high-fat-diet-fed mice [45]. Ethanolic extract of *A. cepa,* including onion leaves, aqueous extract of yellow onion peel, and Welsh onion aqueous/ethanolic extracts, reduced ALT, AST, T-Chol, TG, and LDL levels and improved the HDL level in the blood of high-fat-diet-induced obese Wistar rats and C57BL/6J mice [3,4,46]. Aged black garlic (*A. sativum* L.), aged black elephant garlic (*A. ampeloprasum* L.), and fermented black garlic increased HDL levels and decreased LDL, TG, and cholesterol levels and liver damage by reducing ALT and AST levels, which are major symptoms found in obesity [47,48]. An herbal extract mixed with *A. fistulosum* and *Viola mandshurica* reduced concentrations of glucose, TG, T-Chol, LDL, ALT, and AST and enhanced HDL levels in the serum of obese mice [49]. Aqueous and ethanolic extracts of *A. hookeri* roots controlled levels of ALT, AST, T-Chol, TG, HDL, and LDL in type 2 diabetic mice [36]. In accordance with the results, *A. hookeri* leaf extracts grown under artificial lights positively affected the levels of HDL and LDL. The HDL levels in the blood of mice fed a high-fat diet and *A. hookeri* leaves grown under artificial lights increased by 36.7 to 49.3%, while LDL levels decreased by 0.9 to 32.1% compared with that of the NC group in this study. *A. hookeri* leaf extracts grown in a plant factory with artificial lights may be considered healthy supplements for the recovery of liver injury and improving blood cholesterol and glucose conditions. Also, *A. hookeri* leaves grown in a plant factory with artificial lights can prevent hyperlipemia by reducing levels of T-Chol, TG, and LDL and increasing the level of HDL; thus, they are useful in the treatment of obesity and diabetes.

### 2.7. Effects of A. hookeri Leaf Extracts on the Splenocyte Proliferation in Immunosuppressed Obese Mice

**Figure 4:**
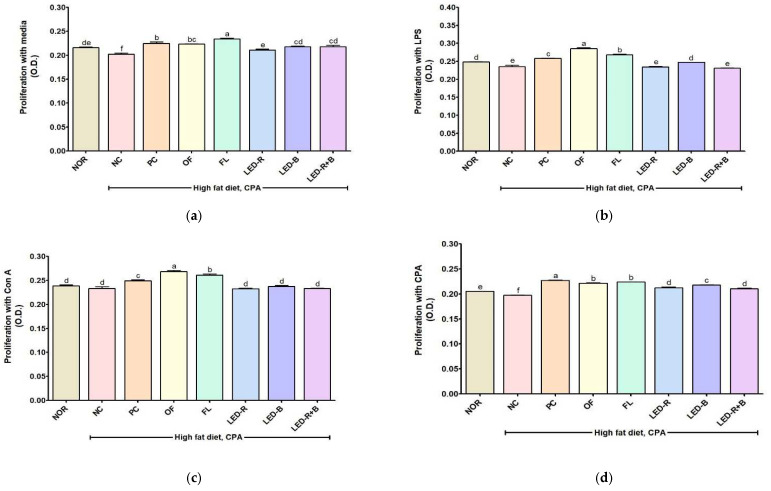
Effects of *A. hookeri* leaf extracts on the proliferation of splenocytes treated with (**a**) media, (**b**) LPA, (**c**) Con A, and (**d**) CPA in obese mice immunosuppressed by CPA. NOR, normal control fed normal diet; immunosuppressed obese groups treated with CPA and high-fat diet. NC, negative control; PC, positive control (β-glucan 50 mg/kg BW); OF, *A. hookeri* leaves grown in outfield (300 mg/kg BW); FL, *A. hookeri* leaves grown under fluorescent lamp (300 mg/kg BW); LED-R, *A. hookeri* leaves grown under LED-R (300 mg/kg BW); LED-B, *A. hookeri* leaves grown under LED-B (300 mg/kg BW); LED-R+B, *A. hookeri* leaves grown under LED-R+B (300 mg/kg BW). Data were presented as the mean ± SEM. ^a–f^ Different letters are significantly different among groups at *p* < 0.05 by Duncan’s multiple range test.

Splenocytes are involved in the initiation of immune reactions against antigens, and the size and number of cells can be used as the immune index [50]. LPS stimulates the proliferation of B cells, whereas Con A stimulates the proliferation of T cells [50]. CPA causes a cytotoxic reaction by forming reactive metabolites that alkylate DNA and proteins, generate cross-links, and inhibit the differentiation of T cells [34]. This study assessed the proliferation of splenocytes to identify the effects of *A. hookeri* leaves grown under artificial lights (Figure 4). The NC group showed the lowest levels of splenocyte proliferation incubated with media, LPS, Con A, and CPA among all immunosuppressed groups. The administration of *A. hookeri* leaf extracts enhanced splenocyte proliferation. Generally, OF and FL groups showed higher levels than those of other immunosuppressed groups. *A. cepa* skin and *S. horneri* improved the proliferation of splenocytes in CPA-induced immunosuppression mice in the presence of LPS and Con A [42,43]. Hydrolyzed and fermented *P. grandiflorum* extract resulted in an increase of splenocyte proliferation in CPA-induced immunosuppressed mice in the treatment of media only, Con A, and LPS [36]. *A. hookeri* leaf extracts improved the proliferation of both T and B cells; thus, *A. hookeri* leaves cultured in the outfield or a plant factory may be used as candidates to enhance the immune system in the immunosuppressed model.

### 2.8. Effects of A. hookeri Leaf Extracts on the NK Cell Activity in Immunosuppressed Obese Mice

**Figure 5:**
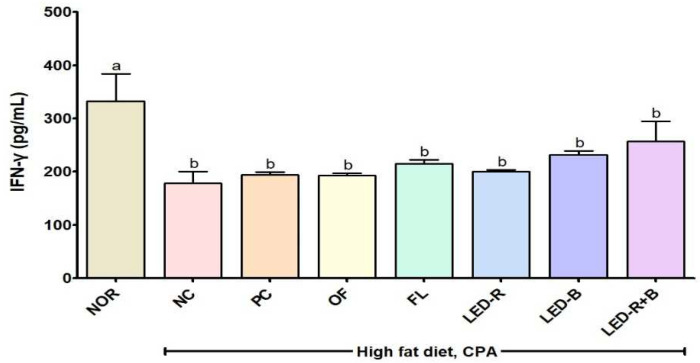
Effects of *A. hookeri* leaf extracts on NK cell activity in blood of immunosuppressed obese mice. NOR, normal control fed normal diet; immunosuppressed obese groups treated with CPA and high-fat diet. NC, negative control; PC, positive control (β-glucan 50 mg/kg BW); OF, *A. hookeri* leaves grown in outfield (300 mg/kg BW); FL, *A. hookeri* leaves grown under fluorescent lamp (300 mg/kg BW); LED-R, *A. hookeri* leaves grown under LED-R (300 mg/kg BW); LED-B, *A. hookeri* leaves grown under LED-B (300 mg/kg BW); LED-R+B, *A. hookeri* leaves grown under LED-R+B (300 mg/kg BW). Data were presented as the mean ± SEM. ^a,b^ Different letters are significantly different among groups at *p* < 0.05 by Duncan’s multiple range test.

NK cell activity was evaluated by measuring the concentration of IFN-γ (Figure 5) because the cells control the immune response by secreting IFN-γ [51]. The concentration of IFN-γ was 332.86 pg/mL in the NOR group and decreased to 178.57 and 193.75 pg/mL in the NC and PC groups. The IFN-γ levels in the blood of immunosuppressed obese mice were 193.22, 214.64, 200.18, 232.14, and 257.14 pg/mL in the OF, FL, LED-R, LED-B, and LED-R+B groups, respectively, and values were higher in groups fed *A. hookeri* leaves cultured in the plant factory, though there was no significant difference found among all immunosuppressed groups. Previous studies investigated that *A. cepa* skin and *P. basummii* improved NK cell activity in the blood, spleen, and peritoneal cells of immunosuppressed mice [42,44]. *A. hookeri* leaves and roots stimulated NK cell activity against Yac-1 in splenocytes of immunosuppressed mice [35]. The mild NK cell activities in the *A. hookeri* leaf groups may be explained with the relatively lower CPA treatment at 100 mg/kg BW by 2 times on −3 and −1 day before the experiment, which were treated at 150 and 100 mg/kg BW in previous trials for the immunosuppressed model. Thus, *A. hookeri* leaves grown in plant factories with artificial lights may be used as immune-enhancing supplements. However, their immune-stimulating effects should be verified in different types of immune-depressed models.

### 2.9. Effects of A. hookeri Leaf Extracts on Serum IgG and Cytokines Levels of Immunosuppressed Obese Mice

**Figure 6:**
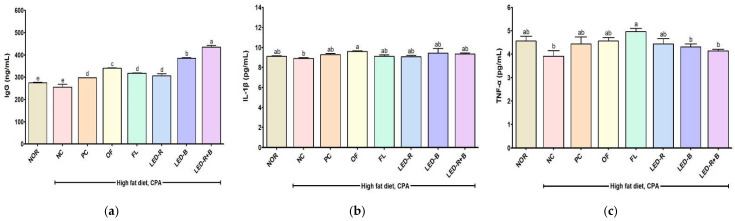
Effects of *A. hookeri* leaf extracts on the serum (**a**) Ig G, (**b**) IL-1β, and (**c**) TNF-α levels in obese mice immunosuppressed by CPA. NOR, normal control fed normal diet; immunosuppressed obese groups treated with CPA and high-fat diet. NC, negative control; PC, positive control (β-glucan 50 mg/kg BW); OF, *A. hookeri* leaves grown in outfield (300 mg/kg BW); FL, *A. hookeri* leaves grown under fluorescent lamp (300 mg/kg BW); LED-R, *A. hookeri* leaves grown under LED-R (300 mg/kg BW); LED-B, *A. hookeri* leaves grown under LED-B (300 mg/kg BW); LED-R+B, *A. hookeri* leaves grown under LED-R+B (300 mg/kg BW). Data were presented as the mean ± SEM. ^a–e^ Different letters are significantly different among groups at *p* < 0.05 by Duncan’s multiple range test.

IgG, IL-1β, and TNF-α levels are shown in Figure 6. IgG, as the major serum immunoglobulin, is principally responsible for the recognition, neutralization, and elimination of pathogens and toxic antigens [52]. Serum IgG level significantly decreased in the NC group compared to the NOR group and increased to 340.67, 318.00, 306.00, 385.67, and 435.50 ng/mL in the OF, FL, LED-R, LED-B, and LED-R+B groups, respectively. IgG levels significantly increased in all *A. hookeri* leaf groups compared to the NC group and were significantly higher than that of the PC group. The LED-R+B group showed the highest IgG level among all experimental mice. In previous studies, *A. hookeri* leaf and root extracts improved the concentrations of IgG [35], and onion skin, fermented *P. grandiflorum*, and aged *P. grandiflorum* extracts also increased serum IgG concentrations in CPA-induced immunosuppressed mice [34,42,53].

Pro-inflammatory cytokines such as IL-1β and TNF-α are mainly produced by activated macrophages and up-regulate the inflammatory reactions [54]. The IL-1β level decreased in the NC group compared to the NOR group but increased in *A. hookeri* leaf groups (*p* > 0.05), and a significant difference was found in the OF group compared to the NC group. TNF-α concentration was lower in the NC group compared to that of the NOR group and was significantly higher in the FL group rather than the NC group. In previous studies, *A. hookeri* leaf and root extracts increased serum IL-1β and TNF-α levels [35], onion skin improved serum IL-1β levels, and *P. grandiflorum* controlled the serum TNF-α levels in CPA-induced immunosuppressed mice, respectively [34,42,53]. In this study, all *A. hookeri* leaf extracts stimulated the production of IgG and cytokines in the immunosuppressed mice (*p* < 0.05 or *p* > 0.05); *A. hookeri* leaves grown in plant factories with artificial lights may be used as immune-enhancing foods.

### 2.10. Effects of A. hookeri Leaf Extracts Grown in Plant Factories with Artificial Lights on MAPK and NF-κB Signaling Pathways in Immunosuppressed Obese Mice

**Figure 7:**
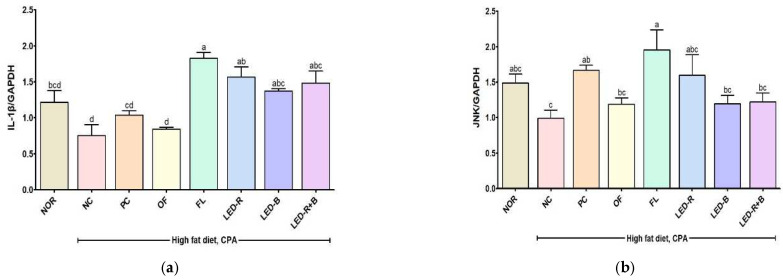

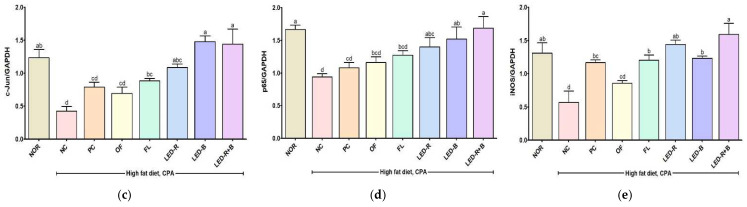
Effects of *A. hookeri* leaf extracts on the protein expressions related to signaling pathways in thymus of CPA-induced immunosuppressed mice. (**a**) IL-1β, MAPKs ((**b**) JNK and (**c**) c-Jun) and NF-κB ((**d**) p65 and (**e**) iNOS). NOR, normal control fed normal diet; immunosuppressed obese groups treated with CPA and high-fat diet. NC, negative control; PC, positive control (β-glucan 50 mg/kg BW); OF, *A. hookeri* leaves grown in outfield (300 mg/kg BW); FL, *A. hookeri* leaves grown under fluorescent lamp (300 mg/kg BW); LED-R, *A. hookeri* leaves grown under LED-R (300 mg/kg BW); LED-B, *A. hookeri* leaves grown under LED-B (300 mg/kg BW); LED-R+B, *A. hookeri* leaves grown under LED-R+B (300 mg/kg BW). Data were presented as the mean ± SEM. ^a–d^ Different letters are significantly different among groups at *p* < 0.05 by Duncan’s multiple range test.

The mitogen-activated protein kinases (MAPK) and nuclear factor kappa-B (NF-κB) signaling pathways play an important role in the immune system [55]. Activated MAPK regulates cell growth, gene expression, and differentiation, and NF-κB is a vital expression factor that regulates genes involved in innate and adaptive immune responses [56]. Figure 7 shows the normalized protein expressions of IL-1β, JNK, c-Jun, p65, and iNOS in the thymus. CPA reduced the expressions of proteins such as IL-1β, JNK, c-Jun, p65, and iNOS in the thymus of immunosuppressed obese mice. However, *A. hookeri* leaves grown in plant factories with artificial lights improved their expressions and increased the levels.

Previous studies reported that the treatment of *Cyclina sinensis* pentadecapeptide raised the protein level of p65 in the spleen of immunosuppressed mice [57], and *Cervus nippon* mantchuricus extract activated iNOS and JNK expressions in immunosuppressed mice [7]. Our results showed similar patterns compared with previous results. The results suggest that *A. hookeri* leaves grown under artificial lights can promote immunomodulatory effects on the immunosuppressed model.

### 2.11. Effects of A. hookeri Leaf Extracts Grown in Plant Factories with Artificial Lights on Superoxide Dismutase and Catalase Activities in Immunosuppressed Obese Mice

**Figure 8:**
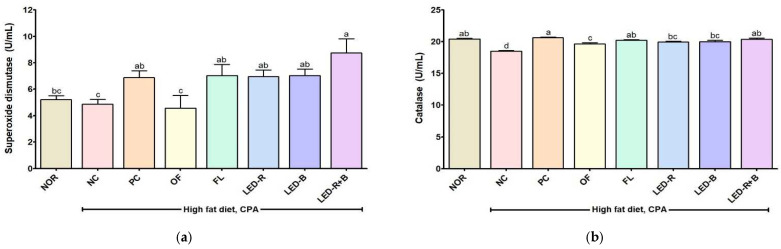
Effects of *A. hookeri* leaf extracts on (**a**) SOD and (**b**) CAT activities in serum of obese mice immunosuppressed by CPA. NOR, normal control fed normal diet; immunosuppressed obese groups treated with CPA and high-fat diet. NC, negative control; PC, positive control (β-glucan 50 mg/kg BW); OF, *A. hookeri* leaves grown in outfield (300 mg/kg BW); FL, *A. hookeri* leaves grown under fluorescent lamp (300 mg/kg BW); LED-R, *A. hookeri* leaves grown under LED-R (300 mg/kg BW); LED-B, *A. hookeri* leaves grown under LED-B (300 mg/kg BW); LED-R+B, *A. hookeri* leaves grown under LED-R+B (300 mg/kg BW). Data were presented as the mean ± SEM. ^a–d^ Different letters are significantly different among groups at *p* < 0.05 by Duncan’s multiple range test.

Superoxide dismutase (SOD) and catalase (CAT) are enzymatic antioxidants. SOD is the first detoxification enzyme and acts as a component of the first-line defense system against reactive oxygen species [58]. CAT is a common antioxidant enzyme in tissues and catalyzes the degradation or reduction of hydrogen peroxide to water [58]. SOD activity was 5.21 U/mL in the NOR group and decreased to 4.86 U/mL in the NC group. The administration of β-glucan improved SOD activity to 6.89 U/mL. SOD activity in the serum of immunosuppressed mice was 4.57, 7.03, 6.96, 7.03, and 8.74 U/mL in the OF, FL, LED-R, LED-B, and LED-R+B groups, respectively (Figure 8). CAT activity was 20.41 U/mL in the NOR group and reduced to 18.53 U/mL in the NC group, while it was increased to 20.64, 19.67, 20.23, 19.94, 20.02, and 20.40 U/mL in the PC, OF, FL, LED-R, LED-B, and LED-R+B groups, respectively. The administration of *A. hookeri* leaves grown in plant factories with artificial lights significantly improved SOD and CAT activities compared with the NC group in the immunosuppressed mice (Figure 8). A previous study reported that *A. hookeri* leaves and roots increased levels of SOD and CAT in RAW 264.7 cells [35]. The administration of onion peel extract and onion powder restored SOD and CAT activities in the liver and kidneys of diabetic rats [59]. This result indicated that *A. hookeri* leaves can eliminate oxidative stress in the body, and *A. hookeri* leaves grown in plant factories with artificial lights can be a useful antioxidant supplement.

### 2.12. Effects of A. hookeri Leaf Extracts Grown in Plant Factories with Artificial Lights on Histopathological Properties in Liver of Immunosuppressed Obese Mice

**Figure 9:**
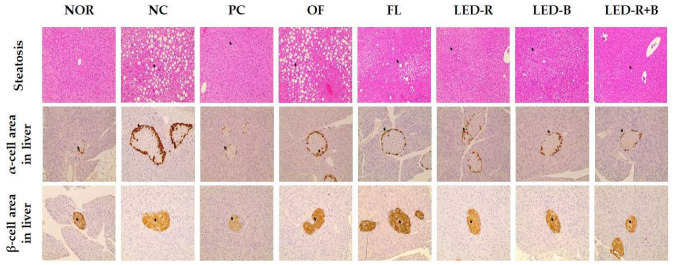
Effects of *A. hookeri* leaf extracts on histopathological analysis of the immunosuppressed obese mice. NOR, normal control fed normal diet; immunosuppressed obese groups treated with CPA and high-fat diet. NC, negative control; PC, positive control (β-glucan 50 mg/kg BW); OF, *A. hookeri* leaves grown in outfield (300 mg/kg BW); FL, *A. hookeri* leaves grown under fluorescent lamp (300 mg/kg BW); LED-R, *A. hookeri* leaves grown under LED-R (300 mg/kg BW); LED-B, *A. hookeri* leaves grown under LED-B (300 mg/kg BW); LED-R+B, *A. hookeri* leaves grown under LED-R+B (300 mg/kg BW).

The effects of *A. hookeri* leaf extracts on the histopathological properties of the immunosuppressed obese mice were investigated and are shown in Figure 9. Steatosis increased in all immunosuppressed obese mice compared with the normal group and decreased in the PC group compared to the NC group. *A. hookeri* leaves grown in a plant factory with artificial lights showed reduced steatosis compared with *A. hookeri* leaves grown in the outfield. In particular, the LED-R+B group alleviated steatosis more than the other groups. A positive brown reaction was observed for glucagon in α-cells and for insulin in β-cells. The stainings α- and β-cells were higher in the NC group compared to the NOR group. The administration of *A. hookeri* leaves reduced brown density compared to the NC group, and the highest effect was found in the LED-R+B group. Similar patterns were found between steatosis and reactions to glucagon and insulin in hepatic α- and β-cells.

**Figure 10:**
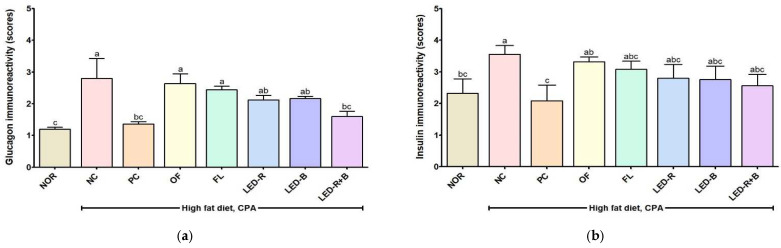
Effects of *A. hookeri* leaf extracts on (**a**) glucagon and (**b**) insulin immunoactivity scores in the liver of the immunosuppressed obese mice. NOR, normal control fed normal diet; immunosuppressed obese groups treated with CPA and high-fat diet. NC, negative control; PC, positive control (β-glucan 50 mg/kg BW); OF, *A. hookeri* leaves grown in outfield (300 mg/kg BW); FL, *A. hookeri* leaves grown under fluorescent lamp (300 mg/kg BW); LED-R, *A. hookeri* leaves grown under LED-R (300 mg/kg BW); LED-B, *A. hookeri* leaves grown under LED-B (300 mg/kg BW); LED-R+B, *A. hookeri* leaves grown under LED-R+B (300 mg/kg BW). Data were presented as the mean ± SEM. ^a–c^ Different letters are significantly different among groups at *p* < 0.05 by Duncan’s multiple range test.

Immunohistochemical results showed that glucagon and insulin responses increased in immunosuppressed obese mice compared with the NOR group (Figure 10). The PC and *A. hookeri* leaf groups showed less immunoreactivity compared with the NC groups. Glucagon and insulin immunoreactivities decreased in the PC and *A. hookeri* leaf groups, and a significant difference in glucagon was found between the LED-R+B and NC groups. Thus, the histopathological results showed the possibility of *A. hookeri* grown in a plant factory with artificial lights relieving the conditions of diabetic patients.

## 3. Materials and Methods

### 3.1. Preparation of Plant Materials

#### 3.1.1. Cultivated Condition

*A. hookeri* was cultivated in hydroponic plant factory within National Institute of Agricultural Sciences. Plants were grown under different experimental setups of FL (24W, Philips, Hamburg, Germany), red LED (LED-R; SungJae CO., Ltd., Sungnam, Korea), blue LED (LED-B; SungJae CO., Ltd.), and mixed red and blue LED (ratio = 1:1; LED-R+B; SungJae CO., Ltd.). Peak wavelengths of R and B were 660 nm and 470 nm, respectively. Two weeks after seeding, light intensity was controlled at 150 μmol/m^2^/s, and temperature and humidity were maintained at 22 ± 1 °C and 50 ± 5%, respectively. *A. hookeri* was cultivated in a deep flow technique hydroponic beds supplemented with Yamazaki nutrient solution. *A. hookeri* cultured in Sunchang, Korea, was used as a control (outfield, OF).

#### 3.1.2. Preparation of Samples

*A. hookeri* leaves were dried in a drying oven at 60 °C and ground using a high-speed mixer (HMF-3260S, Hanil Electric, Gyeonggi, Korea). *A. hookeri* leaves were added with 10-fold volume of 50% (*v*/*v*) ethanol and extracted twice at 160 rpm at room temperature (RT) for 24 h on a shaker (JSOS-500, JSR, Gongju, Korea). Extracts were filtered through filter paper (Whatman No. 6, Cytiva, Marlborough, MA, USA) and were concentrated by rotary evaporator (EYELA N-1000, Riakikai Co., Tokyo, Japan) at 50 °C. The extracts were freeze-dried (PVTFD 10R, Ilsin Lab, Yangju, Korea) and then stored at −80 °C until used.

### 3.2. Evaluation of Functional Compound and Diastatic Enzyme Inhibitory Activity

#### 3.2.1. Alliin and Cycloalliin Concentrations

The extract or standard ((L-alliin, C_6_H_11_NO_3_S, Sigma-Aldrich Co., St. Louis, MO, USA; cycloalliin, C_6_H_11_NO_3_SHCl·H_2_O, Fujifilm Wako Pure Chemical Co., Osaka, Japan) was dissolved in methanol at 0.1 g/mL. Agilent 6410 Triple Quad LC/MS connected to an MS QQQ mass spectrometer with an electrospray ionization source (Agilent Technologies Inc., Santa Clara, CA, USA) was used. Chromatographic separations were performed on a reversed-phase C18 with polar end-capping (150 × 2 mm, SynergiTM 4 μm Hydro-RB 80 Å; Phenomenex, Torrance, CA, USA). The operating temperature was set at 30 °C, the column flow rate was 0.2 mL/min, and the injection volume was 5 μm. Mobile phases A and B consisted of 0.1% formic acid in distilled water and 0.1% formic acid in acetonitrile, respectively. Sample was eluted with the following gradient: 0 min, 5% B; 1 min, 5% B; 11 min, 100% B; 12 min, 100% B; 15 min, 5% B; 20 min, 5% B. The setting conditions were as follows: gas temperature, 300 °C; gas flow, 11 L/min; nebulizer, 15 psi; capillary, 4000 V. MS QQQ mass spectrometer was operated for electrospray ionization (ESI). Detection of the ions was carried out in the multiple-reaction monitoring mode by monitoring the transition pairs of *m*/z 178→73. With the help of a thin-layer chromatography technique and computer-assisted image analysis, we performed the quantitative determination of cycloalliin. The protonated fragments at *m*/*z* of 73 and 178 were observed in the positive ESI-MS/MS spectrum and were consistent with those of the standard compound [35,60].

#### 3.2.2. α-Amylase Inhibition Assay

The inhibition of α-amylase was determined by α-amylase inhibitor screening kit (ab283391, abcam, Cambridge, UK). In brief, 50 μL of samples was mixed with 50 μL of α-amylase enzyme solution at 400 rpm for 1 min. It was incubated at RT for 10 min and protected from light during incubation. The mixture was added with 50 μL of α-amylase substrate and shaken at 400 rpm for 1 min. The absorbance was measured at 405 nm in kinetic mode for 25 min by a microplate reader (Molecular Devices, San Jose, CA, USA). α-Amylase inhibitory activity was calculated by the following formula.

$$\%\;\mathrm{relative}\;\mathrm{inhibition}\;\mathrm{against}\;\alpha \mathrm{-amylase}=\frac{\mathrm{Slope}\;\mathrm{of}\;\mathrm{enzyme}\;\mathrm{control}-\mathrm{Slope}\;\mathrm{of}\;\mathrm{sample}}{\mathrm{Slope}\;\mathrm{of}\;\mathrm{enzyme}\;\mathrm{control}}\;\times \;100$$

#### 3.2.3. α-Glucosidase Inhibition Assay

The inhibition of α-glucosidase was determined by α-glucosidase inhibitor screening kit (ab284520, abcam). The mixture was added with 10 μL sample, 10 μL α-glucosidase enzyme solution, and 60 μL α-glucosidase assay buffer in 96-well plate. The mixture was shaken at 400 rpm for 1 min and incubated at RT for 20 min in dark conditions. The mixture was added with 20 μL reaction mix and shaken at 400 rpm for 1 min. The absorbance was measured at 410 nm in kinetic mode at RT for 60 min by a microplate reader (Molecular Devices). α-Glucosidase inhibitory activity was calculated by the following formula.

$$\%\;\mathrm{relative}\;\mathrm{inhibition}\;\mathrm{against}\;\alpha \mathrm{-glucosidase}=\frac{\mathrm{Slope}\;\mathrm{of}\;\mathrm{enzyme}\;\mathrm{control}-\mathrm{Slope}\;\mathrm{of}\;\mathrm{sample}}{\mathrm{Slope}\;\mathrm{of}\;\mathrm{enzyme}\;\mathrm{control}}\;\times \;100$$

### 3.3. Animal Experiment

#### 3.3.1. Experimental Design

**Figure 11:**
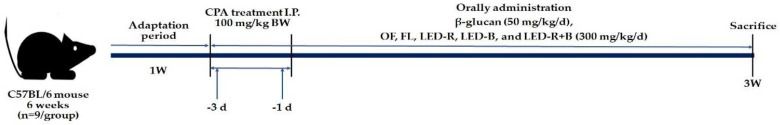
The experimental procedure.

C57BL/6 mice in specific-pathogen-free (SPF) conditions were purchased from Central Lab. Animal Inc. (Seoul, Korea). They were kept in a controlled environment at 23 ± 2 °C with humidity of 50 ± 10% and 12 h light/dark cycle and were fed normal solid feed and water. After one week of acclimation, the mice were fed a normal diet (Research Diets, Inc., New Brunswick, NJ, USA) and a high-fat diet (21% fat, D12079B; Research Diets, Inc.). Cyclophosphamide (CPA; Sigma-Aldrich Co.) was injected intraperitoneally to induce immunosuppression at 100 mg/kg BW before 3 and 1 day(s) of experiment (Figure 11). The weights of the mice were measured after 1 day of 2nd administration with CPA. The mice were randomly divided into 8 groups: (1) normal control (NOR; DW), (2) negative control (NC; DW with CPA), (3) positive control (PC; β-glucan (50 mg/kg BW) with CPA), (4) *A. hookeri* leaves grown in outfield (OF; *A. hookeri* leaves (300 mg/kg BW) with CPA), (5) *A. hookeri* leaves grown in plant factory with FL (FL; *A. hookeri* leaves (300 mg/kg BW) with CPA), (6) *A. hookeri* leaves grown in plant factory with LED-R (LED-R; *A. hookeri* leaves (300 mg/kg BW) with CPA), (7) *A. hookeri* leaves grown in plant factory with LED-B (LED-B; *A. hookeri* leaves (300 mg/kg BW) with CPA), (8) *A. hookeri* leaves grown in plant factory with LED-R+B (LED-R+B; *A. hookeri* leaves (300 mg/kg BW) with CPA). The mice were orally administrated DW, β-glucan, or one of *A. hookeri* leaf extracts for 21 days.

Group 1: NOR (normal control; distilled water, DW).
Group 2: NC (negative control with high-fat diet; CPA; DW).
Group 3: PC (positive control with high-fat diet; CPA; β-glucan 50 mg/kg BW).
Group 4: OF (high-fat diet; CPA; *A. hookeri* leaves grown in outfield, extract 300 mg/kg BW).
Group 5: FL (high-fat diet; CPA; *A. hookeri* leaves grown in plant factory with FL, extract 300 mg/kg BW).
Group 6: LED-R (high-fat diet; CPA; *A. hookeri* leaves grown in plant factory with LED-R, extract 300 mg/kg BW).
Group 7: LED-B (high-fat diet; CPA; *A. hookeri* leaves grown in plant factory with LED-B, extract 300 mg/kg BW).
Group 8: LED-R+B (high-fat diet; CPA; *A. hookeri* leaves grown in plant factory with LED-R+B, extract 300 mg/kg BW).

#### 3.3.2. Determination of Fasting Blood Glucose

Fasting blood glucose was measured from the tail vein using a glucometer (AccuCheck Active, Roche Diagnostics GmbH, Mannheim, Germany) after starvation for 16 h.

#### 3.3.3. Biochemical and Hematological Analysis

Mice were sacrificed under anesthesia (CO_2_) after measuring body weight, and blood was collected from the orbital venous plexus. Whole blood from a mouse was placed in tube with K_2_EDTA and SST (Becton, Dickinson and Company, Franklin Lakes, NJ, USA). SST tube was centrifuged at 1500 rpm and 4 °C for 10 min, and serum was transferred to new tube for biochemical and hematology analysis. HbA1c concentration was measured from the whole blood by the boronate affinity-based method using EasyA1c (Osang Healthcare, Anyang, Korea). The serum levels of total cholesterol (T-Chol), total triglycerides (TG), high-density lipoprotein cholesterol (HDL), low-density lipoprotein cholesterol (LDL), glucose (GLU), alanine aminotransferase (ALT), and aspartate aminotransferase (AST) were measured using a blood biochemical analyzer (7180, HITACHI, Tokyo, Japan). Red blood cell (RBC), hemoglobin (HGB), hematocrit (HCT), mean corpuscular volume (MCV), mean corpuscular hemoglobin (MCH), mean corpuscular hemoglobin concentration (MCHC), platelet (PLT), white blood cell (WBC), neutrophil (NEU), lymphocyte (LYM), monocyte (MONO), eosinophil (EOS), and basophil (BASO) counts were determined by XN-hematology analyzer (Synsmex, Kobe, Japan).

#### 3.3.4. Splenocyte Proliferation

Proliferation assay of splenocytes was determined by a modification of Kim et al. [42]. The spleen was washed by HBSS (GibcoTM, Grand Island, NY, USA) and was homogenized using 40 μm nylon cell strainers (BD Biosciences, San Jose, CA, USA). Spleen cells were centrifuged at 2000 rpm for 10 min to separate lymphocytes. Spleen lymphocytes were seeded at a concentration of 2 × 10^5^ cells/mL into 96-well plates. Concanavalin A (Con A, 1 μg/mL; Sigma-Aldrich Co.), lipopolysaccharide (LPS, 500 ng/mL; Sigma-Aldrich Co.), and CPA (1 μg/mL; Sigma-Aldrich Co.) were added into 96-well plates with splenocytes and were incubated with 5% CO_2_ at 37 °C for 48 h. Media was used as a control. After incubation, 10 μL of MTS solution (Promega Co., Madison, WI, USA) was added and incubated with 5% CO_2_ at 37 °C for 2 h. The absorbance was measured at 490 nm using a microplate reader (Molecular Devices).

#### 3.3.5. NK Cell Activity

Murine NK activity kit (NKMAX Ltd., Seongnam, Gyeonggi, Korea) was used to measure it. Serum was mixed diluent in 96-well plate coated with antibody against murine IFN-γ and incubated at RT for 1 h. Detection antibody conjugate was added into 96-well plate and incubated at RT for 1 h. TMB substrate was mixed with sample and incubated at RT for 30 min in dark conditions. The reaction was stopped with a stop solution. The absorbance was measured at 450 nm using microplate reader (Molecular Devices). IFN-γ concentration was calculated using a standard curve.

#### 3.3.6. Immunoglobulin G (IgG) and Cytokine Concentrations in Serum

Serum IgG concentration was analyzed by IgG ELISA kit (ab283391, abcam, Cambridge, UK) according to kit’s manual. Serum IL-1β and TNF-α concentrations were determined by IL-1β ELISA kit (ab197742, abcam) and TNF-α ELISA kit (ab285327, abcam), respectively, according to the corresponding ELISA kit manual.

#### 3.3.7. Western Blot Analysis Using the Thymus of Immunosuppressed Obese Mice

Total proteins of tissues were extracted by RIPA cell lysis buffer with EDTA (GenDEPOT, Baker, TX, USA) and determined by bicinchoninate assay. After boiling at 100 °C for 5 min, the protein was separated by sodium dodecyl sulfate–polyacrylamide gel electrophoresis (SDS-PAGE) using 4–15% mini-protean TGX gels (BIO-RAD, Hercules, CA, USA) at voltage of 200 V for 30 min. It was transferred to 0.2 μm polyvinylidene difluoride (PVDF) membrane (BIO-RAD). The PVDF membrane was blocked with blocking buffer (BIOMAX, Guri, Gyeonggi, Korea) for 5 min to block non-specific binding at RT and incubated with primary antibodies at 4 °C overnight. The membranes were rinsed with TBST 3 times to wash away the unconjugated primary antibodies. After incubation with horseradish peroxidase (HRP), the secondary antibodies were conjugated at RT for 2 h. The protein immune complexes were prepared with enhanced chemiluminescence (ECL; West-1 Pico Dura ECL Solution, GenDEPOT) and visualized by ChemiDoc^TM^ MP Imaging System with Image Lab^TM^ Software (ver.6.1, BIO-RAD).

#### 3.3.8. Superoxide Dismutase and Catalase in Serum

Superoxide dismutase (SOD) and catalase (CAT) activities were determined by superoxide dismutase (SOD) colorimetric activity kit (EIASODC, Invitrogen, ThermoFisher Scientific, Carlsbad, CA, USA) and catalase colorimetric activity kit (EIACATC, Invitrogen), respectively, according to each kit’s manual.

#### 3.3.9. Histopathological and Immunohistochemical Investigations

Liver tissues were cut into small pieces and immersed in 10% formalin solution at room temperature for 24 h. The fixed tissues were embedded in paraffin and cut into 3 μm sections using a Bond Polymer Intense Detection System (Vision BioSystems, Melbourne, Australia). The sectioned tissue was pre-treated, double-stained with hematoxylin and eosin, and subjected to periodic acid–Schiff (PAS) reaction, followed by dehydration. It was observed under an optical microscope at 200× magnification and photographed with a camera attached to a microscope [36].

### 3.4. Statistical Analysis

All samples were carried out in triplicate and analyzed using one-way analysis of variance followed by Duncan’s multiple range test (version 18.0, SPSS Inc., Chicago, IL, USA). Data were expressed as mean ± SEM, and values were considered statistically significant at *p* < 0.05.

## 4. Conclusions

All *A. hookeri* leaf extracts positively affected the immune system, biochemical parameters, and toxicological factors in immunosuppressed obese mice. *A. hookeri* leaves increased the immune-related organ (spleen and thymus) index; the counts of RBC, PLT, WBC, and LYM; HDL concentrations; and the proliferation of splenocytes, IgG, and cytokine (IL-1β and TNF-α) levels. *A. hookeri* leaves also effectively reduced diabetes- and obese-related organ (liver, heart, epididymal fat, and pancreas) indexes, fasting blood glucose concentration, ALT and AST levels, and lipid (T-Chol, TG, and LDL) and GLU values. *A. hookeri* leaves grown in the plant factory with artificial lights (FL, LED-R, -B, and -R+B) showed higher inhibitory activity against α-amylase and α-glucosidase in in vitro than *A. hookeri* leaves grown outfield. Increased expressions of IL-1β, JNK, c-Jun, p65, and iNOS were found in the thymus of the mice supplemented with *A. hookeri* leaves grown in the plant factory. The results suggested that artificial lights used in this study may culture *A. hookeri* leaves as plants with high immunomodulatory and anti-diabetic effects. *A. hookeri* leaves grown in plant factories with artificial lights may also be used as novel functional foods improving public health in immunosuppressed and obese conditions.

## Figures and Tables

**Table 1:** Alliin and cycloalliin concentrations of *A. hookeri* leaves grown in the plant factory with artificial lights.

Sample	Alliin		Cycloallin	
	Peak Area	Concentration (μg/g)	Peak Area	Concentration (μg/g)
OF ^1^	3,817,751	1669.66	697,544	442.20
FL	458,234	200.19	227,163	143.77
LED-R	269,700	117.72	159,380	100.77
LED-B	162,735	70.93	103,263	65.17
LED-R+B	260,231	113.58	158,170	100.00

^1^ OF, *A. hookeri* leaves grown in outfield; FL, *A. hookeri* leaves grown in the plant factory with fluorescent lamp; LED-R, *A. hookeri* leaves grown in the plant factory with red LEDs; LED-B, *A. hookeri* leaves grown in the plant factory with blue LEDs; LED-R+B, *A. hookeri* leaves grown in the plant factory with red + blue LEDs.

**Table 2:** Effects of *A. hookeri* leaf extracts on the body and organ weights of the immunosuppressed obese mice.

	NOR ^1^	Immunosuppressed Obese Mice ^2^						
		NC	PC	OF	FL	LED-R	LED-B	LED-R+B
Initial body weight (g)	21.27 ± 0.28 ^ns^	21.37 ± 0.12	21.17 ± 0.12	21.40 ± 0.35	21.07 ± 0.15	21.00 ± 0.31	21.00 ± 0.10	21.47 ± 0.03
Final body weight (g)	25.43 ± 0.57 ^ab^	23.90 ± 0.22 ^c^	26.33 ± 0.45 ^a^	26.28 ± 0.21 ^a^	25.30 ±0.41 ^ab^	25.10 ± 0.35 ^b^	24.95 ± 0.33 ^bc^	25.08 ± 0.14 ^b^
Tissue weight (% of BW)								
Spleen	0.26 ± 0.01 ^ab^	0.23 ± 0.01 ^b^	0.30 ± 0.01 ^a^	0.30 ± 0.01 ^a^	0.30 ± 0.02 ^a^	0.27 ± 0.00 ^a^	0.28 ± 0.01 ^a^	0.28 ± 0.01 ^a^
Thymus	0.22 ± 0.02 ^a^	0.13 ± 0.01 ^c^	0.20 ± 0.01 ^ab^	0.20 ± 0.01 ^ab^	0.17 ± 0.02 ^bc^	0.19 ± 0.02 ^ab^	0.19 ± 0.00 ^ab^	0.20 ± 0.00 ^ab^
Liver	4.28 ± 0.29 ^b^	4.72 ± 0.10 ^a^	4.25 ± 0.14 ^b^	4.39 ± 0.07 ^ab^	4.36 ± 0.08 ^ab^	4.38 ± 0.08 ^ab^	4.10 ± 0.13 ^b^	4.19 ± 0.02 ^b^
Heart	0.46 ± 0.01 ^b^	0.52 ± 0.01 ^a^	0.46 ± 0.00 ^b^	0.46 ± 0.01 ^b^	0.46 ± 0.02 ^b^	0.47 ± 0.01 ^b^	0.46 ± 0.01 ^b^	0.47 ± 0.00 ^b^
Epididymal fat	1.27 ± 0.04 ^c^	2.35 ± 0.27 ^a^	1.37 ± 0.05 ^bc^	1.52 ± 0.07 ^bc^	1.29 ± 0.07 ^c^	1.51 ± 0.07 ^bc^	1.49 ± 0.09 ^bc^	1.71 ± 0.13 ^b^
Pancreas	1.06 ± 0.04 ^c^	1.87 ± 0.10 ^a^	1.71 ± 0.05 ^ab^	1.73 ± 0.04 ^ab^	1.77 ± 0.08 ^ab^	1.69 ± 0.02 ^b^	1.77 ± 0.04 ^ab^	1.69 ± 0.03 ^b^

^1^ NOR, normal control fed normal diet. ^2^ Immunosuppressed obese groups treated with CPA and high-fat diet. NC, negative control; PC, positive control (β-glucan 50 mg/kg BW); OF, *A. hookeri* leaves grown in outfield (300 mg/kg BW); FL, *A. hookeri* leaves grown under fluorescent lamp (300 mg/kg BW); LED-R, *A. hookeri* leaves grown under LED-R (300 mg/kg BW); LED-B, *A. hookeri* leaves grown under LED-B (300 mg/kg BW); LED-R+B, *A. hookeri* leaves grown under LED-R+B (300 mg/kg BW). Data were expressed as the mean ± SEM. ^ns^ No significance. ^a–c^ Different letters are significantly different among groups at *p* < 0.05 by Duncan’s multiple range test.

**Table 3:** Effects of *A. hookeri* leaf extracts on hematological factors of immunosuppressed obese mice.

			NOR ^1^	Immunosuppressed Obese Mice ^2^						
				NC	PC	OF	FL	LED-R	LED-B	LED-R+B
RBC (×10^6^ cells/μL) ^3^			10.76 ± 0.23 ^a^	9.68 ± 0.05 ^e^	10.22 ± 0.09 ^cd^	10.18 ± 0.14 ^cd^	10.00 ± 0.04 ^de^	10.62 ± 0.15 ^ab^	10.46 ± 0.12 ^abc^	10.33 ± 0.05 ^bcd^
HGB (g/dL)			15.60 ± 0.30 ^a^	14.17 ± 0.13 ^d^	14.50 ± 0.15 ^cd^	14.80 ± 0.26 ^bc^	14.73 ± 0.12 ^cd^	15.40 ± 0.25 ^ab^	15.00 ± 0.12 ^abc^	14.97 ± 0.09 ^bc^
HCT (%)			56.93 ± 1.41 ^a^	46.10 ± 0.17 ^d^	50.47 ± 0.20 ^c^	53.40 ± 0.76 ^bc^	52.37 ± 0.91 ^bc^	53.93 ± 1.55 ^b^	52.57 ± 0.90 ^bc^	51.80 ± 0.66 ^bc^
RBC Index	MCV (fL)		54.10 ± 0.46 ^a^	46.37 ± 0.27 ^d^	51.73 ± 0.09 ^c^	53.47 ± 0.41 ^ab^	53.43 ± 0.23 ^ab^	52.67 ± 0.35 ^abc^	52.40 ± 0.96 ^bc^	51.50 ± 0.31 ^c^
	MCH (pg)		14.30 ± 0.10 ^b^	14.83 ± 0.13 ^a^	14.10 ± 0.25 ^b^	14.33 ± 0.03 ^b^	14.37 ± 0.03 ^b^	14.47 ± 0.03 ^b^	14.20 ± 0.10 ^b^	14.27 ± 0.09 ^b^
	MCHC (g/dL)		27.17 ± 0.09 ^b^	31.53 ± 0.18 ^a^	27.47 ± 0.39 ^b^	27.20 ± 0.26 ^b^	27.53 ± 0.18 ^b^	27.43 ± 0.24 ^b^	27.57 ± 0.32 ^b^	27.60 ± 0.35 ^b^
PLT (×10^3^ cells/μL)			1803.33 ± 110.09 ^a^	1100.00 ± 16.92 ^c^	1336.67 ± 16.02 ^b^	1254.33 ± 15.30 ^b^	1263.67 ± 12.03 ^b^	1278.33 ± 22.82 ^b^	1279.33 ± 14.15 ^b^	1209.00 ± 53.98 ^bc^
WBC (×10^3^ cells/μL)			3.74 ± 0.06 ^a^	1.51 ± 0.17 ^c^	2.44 ± 0.36 ^b^	2.66 ± 0.05 ^b^	2.11 ± 0.03 ^bc^	2.65 ± 0.29 ^b^	2.68 ± 0.16 ^b^	2.58 ± 0.60 ^b^
WBC Differential Counting (%)		NEU	3.57 ± 0.73 ^d^	8.97 ± 0.90 ^a^	7.80 ± 0.53 ^ab^	4.67 ± 0.13 ^cd^	6.23 ± 0.60 ^bc^	6.00 ± 0.67 ^bc^	6.20 ± 0.45 ^bc^	5.00 ± 0.25 ^cd^
		LYM	91.77 ± 1.23 ^a^	72.13 ± 2.20 ^b^	86.90 ± 1.22 ^a^	89.70 ± 1.55 ^a^	89.40 ± 1.29 ^a^	90.37 ± 0.67 ^a^	86.80 ± 2.71 ^a^	89.73 ± 0.50 ^a^
		MONO	3.10 ± 0.56 ^ab^	2.40 ± 0.15 ^b^	3.33 ± 0.19 ^ab^	3.40 ± 0.46 ^ab^	3.97 ± 0.37 ^a^	3.93 ± 0.09 ^a^	3.83 ± 0.30 ^a^	3.23 ± 0.49 ^ab^
		EOS	0.70 ± 0.10 ^b^	1.87 ± 0.58 ^a^	0.90 ± 0.17 ^b^	0.60 ± 0.06 ^b^	0.70 ± 0.12 ^b^	0.67 ± 0.12 ^b^	0.97 ± 0.09 ^b^	1.23 ± 0.13 ^ab^
		BASO	0.00 ± 0.00 ^b^	0.20 ± 0.10 ^a^	0.00 ± 0.00 ^b^	0.00 ± 0.00 ^b^	0.00 ± 0.00 ^b^	0.00 ± 0.00 ^b^	0.00 ± 0.00 ^b^	0.00 ± 0.00 ^b^

^1^ NOR, normal control fed normal diet; ^2^ immunosuppressed obese groups treated with CPA and high-fat diet. NC, negative control; PC, positive control (β-glucan 50 mg/kg BW); OF, *A. hookeri* leaves grown in outfield (300 mg/kg BW); FL, *A. hookeri* leaves grown under fluorescent lamp (300 mg/kg BW); LED-R, *A. hookeri* leaves grown under LED-R (300 mg/kg BW); LED-B, *A. hookeri* leaves grown under LED-B (300 mg/kg BW); LED-R+B, *A. hookeri* leaves grown under LED-R+B (300 mg/kg BW). ^3^ RBC, red blood cell; HGB, hemoglobin; HCT, hematocrit; MCV, mean corpuscular volume; MCH, mean corpuscular hemoglobin; MCHC, mean corpuscular hemoglobin concentration; PLT, platelet; WBC, white blood cell; NEU, neutrophils; LYM, lymphocytes; MONO, monocytes; EOS, eosinophils; BASO, basophils. Data were expressed as the mean ± SEM. ^a–e^ Different letters are significantly different among groups at *p* < 0.05 by Duncan’s multiple range test.

**Table 4:** Effects of *A. hookeri* leaf extracts on biochemical factors in blood of immunosuppressed obese mice.

	NOR ^1^	Immunosuppressed Obese Mice ^2^						
		NC	PC	OF	FL	LED-R	LED-B	LED-R+B
ALT (U/mL) ^3^	4.80 ± 1.29 ^b^	21.33 ± 6.86 ^a^	6.40 ± 2.81 ^b^	3.33 ± 0.48 ^b^	5.73 ± 1.96 ^b^	4.00 ± 0.23 ^b^	5.40 ± 2.39 ^b^	2.13 ± 0.13 ^b^
AST (U/mL)	36.00 ± 1.89 ^b^	95.73 ± 33.39 ^a^	39.33 ± 1.48 ^b^	39.87 ± 2.36 ^b^	41.47 ± 2.43 ^b^	41.87 ± 3.58 ^b^	48.73 ± 3.28 ^b^	40.93 ± 1.39 ^b^
T-Chol (mg/dL)	106.67 ± 3.53 ^b^	148.00 ± 6.93 ^a^	148.00 ± 0.00 ^a^	134.67 ± 8.11 ^ab^	120.00 ± 28.38 ^ab^	128.00 ± 2.31 ^ab^	136.67 ±11.33 ^ab^	144.00 ± 0.00 ^ab^
TG (mg/dL)	37.33 ± 3.53 ^a^	37.33 ± 6.67 ^a^	18.67 ± 3.53 ^b^	20.00 ± 2.31 ^b^	14.67 ± 3.53 ^b^	20.00 ± 6.93 ^b^	17.33 ± 3.53 ^b^	18.67 ± 4.81 ^b^
HDL (mg/dL)	73.07 ± 2.15 ^ab^	59.20 ± 19.69 ^b^	88.27 ± 5.47 ^a^	85.73 ± 3.10 ^a^	85.60 ± 5.06 ^a^	80.93 ± 3.58 ^ab^	88.40 ± 1.29 ^a^	83.60 ± 0.83 ^ab^
LDL (mg/dL)	5.47 ± 0.13 ^b^	14.53 ±1.87 ^a^	13.73 ± 0.67 ^a^	10.40 ± 1.80 ^a^	9.87 ± 2.92 ^a^	11.33 ± 0.48 ^a^	13.80 ± 0.76 ^a^	14.40 ± 0.40 ^a^
GLU (mg/dL)	317.33 ± 9.61 ^ab^	384.00 ± 50.12 ^a^	306.67 ± 11.62 ^ab^	309.33 ± 30.05 ^ab^	273.33 ± 55.44 ^b^	270.67 ± 24.69 ^b^	250.00 ± 11.02 ^b^	270.67 ± 13.92 ^b^
HbA1c (%)	4.23 ± 0.06 ^ns^	4.14 ± 0.02	4.14 ± 0.02	4.15 ± 0.05	4.10 ± 0.00	4.13 ± 0.03	4.18 ± 0.02	4.13 ± 0.03

^1^ NOR, normal control fed normal diet; ^2^ immunosuppressed obese groups treated with CPA and high-fat diet. NC, negative control; PC, positive control (β-glucan 50 mg/kg BW); OF, *A. hookeri* leaves grown in outfield (300 mg/kg BW); FL, *A. hookeri* leaves grown under fluorescent lamp (300 mg/kg BW); LED-R, *A. hookeri* leaves grown under LED-R (300 mg/kg BW); LED-B, *A. hookeri* leaves grown under LED-B (300 mg/kg BW); LED-R+B, *A. hookeri* leaves grown under LED-R+B (300 mg/kg BW). ^3^ ALT, alanine aminotransferase; AST, aspartate aminotransferase; T-Chol, total cholesterol; TG, triglyceride; HDL, high-density lipoprotein; LDL, low-density lipoprotein; GLU, glucose; HbA1c, hemoglobin A1c. Data were expressed as the mean ± SEM. ^a,b^ Different letters are significantly different at *p* < 0.05 by Duncan’s multiple range test. ^ns^ No significance.

## Data Availability

Data are contained in the article.

## References

[1] Koh Y.M., Jang S.W., Ahn T.W. (2019). Anti-obesity effect of Yangkyuksanwhatang in high-fat diet-induced obese mice. BMC Complement. Altern. Med..

[2] Jee Y.M. (2020). Korea Health Statistics 2021: Korea National Health and Nutrition Examination Survey (KNHANES VIII-3).

[3] Sung Y.Y., Kim D.S., Kim S.H., Kim H.K. (2018). Aqueous and ethanolic extracts of welsh onion, *Allium fistulosum*, attenuate high-fat diet-induced obesity. BMC Complement. Altern. Med..

[4] Yu S., Li H., Cui T., Cui M., Piao C., Wang S., Ju M., Liu X., Zhou G., Xu H. (2021). Onion (*Allium cepa* L.) peel extract effects on 3T3-L1 adipocytes and high-fat diet-induced obese mice. Food Biosci..

[5] Klein S., Gastaldelli A., Yki-Järvinen H., Scherer P.E. (2022). Why does obesity cause diabetes?. Cell Metab..

[6] Marti A., Marcos A., Martínez J.A. (2001). Obesity and immune function relationships. Obes. Rev..

[7] Hong S.H., Ku J.M., Kim H.I., Ahn C.W., Park S.H., Seo H.S., Shin Y.C., Ko S.G. (2017). The immune-enhancing activity of *Cervus nippon mantchuricus* extract (NGE) in RAW264.7 macrophage cells and immunosuppressed mice. Food Res. Int..

[8] Zhang H., Gao J., Tang Y., Jin T., Tao J. (2023). Inflammasomes cross-talk with lymphocytes to connect the innate and adaptive immune response. J. Adv. Res..

[9] Marshall J.S., Warrington R., Watson W., Kim H.L. (2018). An introduction to immunology and immunopathology. Allergy Asthma Clin. Immunol..

[10] Bonilla F.A., Oettgen H.C. (2010). Adaptive immunity. J. Allergy Clin. Immunol..

[11] Tong T., Wang Y.N., Zhang C.M., Kang S.G. (2021). In vitro and in vivo antihypertensive and antioxidant activities of fermented roots of *Allium hookeri*. Chin. Herb. Med..

[12] Deka B., Manna P., Borah J.C., Talukdar N.C. (2022). A review on phytochemical, pharmacological attributes and therapeutic uses of *Allium hookeri*. Phytomed. Plus.

[13] Kim J.S. (2020). Effects of solvents with different polarities on the antioxidant activities of the leaves and roots of *Allium hookeri*. J. East. Asian Soc. Diet. Life.

[14] Jang J.Y., Lee M.J., You B.R., Jin J.S., Lee S.H., Yun Y.R., Kim H.J. (2017). *Allium hookeri* root extract exerts anti-inflammatory effects by nuclear factor-κB down-regulation in lipopolysaccharide-induced RAW264.7 cells. BMC Complement. Altern. Med..

[15] Kim H.J., Lee M.J., Jang J.Y., Lee S.H. (2019). *Allium hookeri* root extract inhibits adipogenesis by promoting lipolysis in high fat diet-induced obese mice. Nutrients.

[16] Chun H., Han S. (2021). A study on the design and operation method of plant factory using artificial intelligence. Nanotechnol. Environ. Eng..

[17] Arcel M.M., Lin X., Huang J., Wu J., Zheng S. (2021). The application of LED illumination and intelligent control in plant factory, a new direction for modern agriculture: A review. J. Phys. Conf. Ser..

[18] Chen X.L., Guo W.Z., Xue X.Z., Wang L.C., Qiao X.J. (2014). Growth and quality responses of ‘green oak leaf’ lettuce as affected by monochromic or mixed radiation provided by fluorescent lamp (FL) and light-emitting diode (LED). Sci. Hortic..

[19] Ma Y., Xu A., Cheng Z.M. (2021). Effects of light emitting diode lights on plant growth, development and traits a meta-analysis. Hortic. Plant J..

[20] Razzak M.A., Asaduzzaman M., Tanaka H., Asao T. (2022). Effects of supplementing green light to red and blue light on the growth and yield of lettuce in plant factories. Sci. Hortic..

[21] Kim Y.M., Sung J.K., Lee Y.J., Lee D.B., Yoo C.H., Lee S.B. (2019). Varying effects of artificial light on plant functional metabolites. Korean J. Environ. Agric..

[22] Cho J.Y., Yoo K.S., Kim J., Choi B.J., Oh W. (2020). Growth and bioactive compounds of lettuce as affected by light intensity and photoperiod in a plant factory using external electrode fluorescent lamps. Hortic. Sci. Technol..

[23] Jung J., Heo J.W., Kim J.S., Jeong U.Y., Bae U.J., Jang H.N., Shim C.K., Joung Y., Lee S.H. (2022). Functionality of *Allium hookeri* leaves and roots grown in a hydroponic plant factory using artificial lights. J. Korean Soc. Food Sci. Nutr..

[24] Jung J., Heo J.W., Kim J.S., Jeong U.J., Kim H.B., Shim C.K., Lee S.H. (2022). Comparison of the antioxidant and anti-inflammatory effects of *Allium hookeri* leaves grown in an outfield and a plant factory using different artificial lights. Korean J. Community Living Sci..

[25] Park S.H., Bae U.J., Choi E.K., Jung S.J., Lee S.H., Yang J.H., Kim Y.S., Jeong D.Y., Kim H.J., Park B.H. (2020). A randomized, double-blind, placebo-controlled crossover clinical trial to evaluate the anti-diabetic effects of *Allium hookeri* extract in the subjects with prediabetes. BMC Complement. Med. Ther..

[26] Lee H.J., Suh H.J., Han S.H., Hong J., Choi H.S. (2016). Optimization of extraction of cycloalliin from garlic (*Allium sativum* L.) by using principal components analysis. Prev. Nutr. Food Sci..

[27] Kalita D., Holm D.G., LaBarbera D.V., Petrash J.M., Jayanty S.S. (2018). Inhibition of α-glucosidase, α-amylase, and aldose reductase by potato polyphenolic compounds. PLoS ONE.

[28] Singh M., Thrimawithana T., Shukla R., Adhikari B. (2022). Inhibition of enzymes associated with obesity by the polyphenol-rich extracts of *Hibiscus sabdariffa*. Food Biosci..

[29] Wu Y.X., Kim Y.J., Li S., Yun M.C., Yoon J.M., Kim J.Y., Cho S.I., Son K.H., Kim T. (2015). Anti-obese effects of mulberry (*Morus alba* L.) root bark through the inhibition of digestive enzymes and 3T3-L1 adipocyte differentiation. Korean J. Food Preserv..

[30] Unuofin J.O., Otunola G.A., Afolayan A.J. (2018). In vitro α-amylase, α-glucosidase, lipase inhibitory and cytotoxic activities of tuber extracts of *Kedrostis africana* (L.) Cogn. Heliyon.

[31] Ardeshirlarijani E., Namazi N., Jalili R.B., Saeedi M., Imanparast S., Adhami H.R., Faramarzi M.A., Ayati M.H., Mahdavi M., Larijani B. (2019). Potential anti-obesity effects of some medicinal herb: In vitro α-amylase, α-glucosidase and lipase inhibitory activity. Int. Biol. Biomed. J..

[32] Kurniawan H., Dacamis E.S., Simamora A., Tobing P.S.D.L., Hanapiah A., Santoso A.W. (2020). Antioxidant, antidiabetic, and anti-obesity potential of *Ipomoea reptans* poir leaves. Borneo J. Pharm..

[33] Vo T.N., Luong T.D.M., Le T.P.H., Trinh K.S. (2022). Control of obesity, blood glucose, and blood lipid with *Olax imbricate* Roxb. root extract in high-fat diet-induced obese mice. J. Toxicol..

[34] Lee H.S., Kim S.M., Jung J.I., Lim J., Woo M., Kim E.J. (2023). Immune-enhancing effect of hydrolyzed and fermented *Platycodon grandiflorum* extract in cyclophosphamide-induced immunosuppressed BALB/c mice. Nutr. Res. Pract..

[35] Jeong U.Y., Jung J., Lee E.B., Choi J.H., Kim J.S., Jang H.H., Park S.Y., Lee S.H. (2022). Antioxidant and immune stimulating effects of *Allium hookeri* extracts in the RAW 264.7 cells and immune-depressed C57BL/6 mice. Antioxidants.

[36] Choi J.H., Kim S.H., Lee E.B., Kim J.S., Jung J.E., Jeong U.Y., Kim J.H., Jang H.H., Park S.Y., Kim G.C. (2022). Anti-diabetic effects of *Allium hookeri* extracts prepared by different methods in type 2 C57BL/J-*db*/*db* mice. Pharmaceuticals.

[37] Qi Q., Dong Z., Sun Y., Li S., Zhao Z. (2018). Protective effect of bergenin against cyclophosphamide-induced immunosuppression by immunomodulatory effect and antioxidation in Balb/c mice. Molecules.

[38] Kim H., Kim J.W., Kim Y.K., Ku S.K., Lee H.J. (2022). Immunoenhancement effects of the herbal formula hemomine on cyclophosphamide-induced immunosuppression in mice. Appl. Sci..

[39] Park Y.H., An M., Kim J.K., Lim Y.H. (2020). Antiobesity effect of ethanolic extract of *Ramulus mori* in differentiated 3T3-L1 adipocytes and high-fat diet-induced obese mice. J. Ethnopharmacol..

[40] Ülger T.G., Çakiroglu F.P. (2020). The effects of onion (*Allium cepa* L.) dried by different heat treatments on plasma lipid profile and fasting blood glucose level in diabetic rats. Avicenna J. Phytomed.

[41] Silva-Santana G., Bax J.C., Fernandes D.C.S., Bacellar D.T.L., Hooper C., Dias A.A.S.O., Silva C.B., Souza A.M., Ramos S., Santos R.A. (2020). Clinical hematological and biochemical parameters in Swiss, BALB/c, C57BL/6 and B6D2F1 *Mus musculus*. Anim. Models Exp. Med..

[42] Kim J.S., Lee E.B., Choi J.H., Jung J., Jeong U.Y., Bae U.J., Jang H.H., Park S.Y., Cha Y.S., Lee S.H. (2023). Antioxidant and immune stimulating effects of *Allium cepa* skin in the RAW 264.7 cells and in the C57BL/6 mouse immunosuppressed by cyclophosphamide. Antioxidants.

[43] Kim H.I., Kim D.S., Jung Y., Sung N.Y., Kim M., Han I.J., Nho E.Y., Hong J.H., Lee J.K., Boo M. (2022). Immune-enhancing effect of *Sargassum horneri* on cyclophosphamide-induced immunosuppression in BALB/c mice and primary cultured splenocytes. Molecules.

[44] Yoo J.H., Lee Y.S., Ku S.K., Lee H.J. (2020). *Phenllius baumii* enhances the immune response in cyclophosphamide-induced immunosuppressed mice. Nutr. Res..

[45] Park S., No K., Lee J. (2018). Anti-obesity effect of *Allium hookeri* leaf extract in high-fat diet-fed mice. J. Med. Food.

[46] Momoh B.J., Okere S.O., Anyanwu G.O. (2022). The anti-obesity effect of *Allium cepa* L. leaves on high fat diet induced obesity in male Wistar rats. Clin. Complement. Med. Pharmacol..

[47] Chae J., Lee E., Oh S.M., Ryu H.W., Kim S., Nam J.O. (2023). Aged black garlic (*Allium sativum* L.) and aged black elephant garlic (*Allium ampeloprasum* L.) alleviate obesity and attenuate obesity-induced muscle atrophy in diet-induced obese C57BL/6 mice. Biomed. Pharmacother..

[48] Jung Y.M., Lee S.H., Lee D.S., You M.J., Chung I.K., Cheon W.H., Kwon Y.S., Lee Y.J., Ku S.K. (2011). Fermented garlic protects diabetic, obese mice when fed a high-fat diet by antioxidant effects. Nut. Res..

[49] Sung Y.Y., Kim S.H., Yoo B.W., Kim H.K. (2015). The nutritional composition and anti-obesity effects of an herbal mixed extract containing *Allium fistulosum* and *Viola mandshurica* in high-fat-diet-induced obese mice. BMC Complement. Altern. Med..

[50] Ryu D.S., Kim S.H., Lee D.S. (2009). Effect of *Salicornia herbacea* polysaccharides on the activation of immune cells in vitro and in vivo. Food Sci. Biotechnol..

[51] Lee J., Park K.H., Ryu J.H., Bae H.J., Choi A., Lee H., Lim J., Han K., Park C.H., Jung E.S. (2017). Natural killer cell activity for IFN-gamma production as a supportive diagnostic marker for gastric cancer. Oncotarget.

[52] Kaneko Y., Nimmerjahn F., Ravetch J.V. (2006). Anti-inflammatory activity of immunoglobulin G resulting from Fc sialylation. Science.

[53] Choi J.H., Lee E.B., Park Y.G., Lee H.K., Jang H.H., Choe J., Hwang K.A., Park S.Y., Hwang I.G., Hong H.C. (2019). Aged doraji (*Platycodon grandiflorum*) ameliorates cyclophosphamide-induced immunosuppression in mice. Korean J. Pharmacogn..

[54] Pal P.P., Begum A.S., Basha S.A., Araya H., Fujimoto Y. (2023). New natural pro-inflammatory cytokines (TNF-α, IL-6 and IL-1β) and iNOS inhibitors identified from *Penicillium polonicum* through in vitro and in vivo studies. Int. Immunopharmacol..

[55] Seo H.J., Jeong J.B. (2020). Immune-enhancing effects of green lettuce (*Lactuca sativa* L.) extracts through the TLR4-MAPK/NF-κB signaling pathways in RAW264.7 macrophage cells. Korean J. Plant Res..

[56] Kim S.J., Shin M.S., Kim M., Baek S.H., Kang K.S. (2021). Characterization of an immune-enhancing polysaccharide fraction isolated from heat-processed ginseng derived from *Panax ginseng* C.A. Meyer. Appl. Sci..

[57] Zhao R., Jiang X.X., Zhao Q.L., Ye H.W., Lin Y., Huang J., Tang Y.P. (2022). Immunoenhancing effects of *Cyclina sinensis* pentadecapeptide through modulation of signaling pathways in mice with cyclophosphamide-induced immunosuppression. Mar. Drugs.

[58] Ighodaro O.M., Akinloye O.A. (2018). First line defence antioxidants-superoxide dismutase (SOD), catalase (CAT) and glutathione peroxidase (GPS): Their fundamental role in the entire antioxidant defence grid. Alex. J. Med..

[59] Masood S., Rehman A., Bashir S., Shazly M.E., Imran M., Khalil P., Ifthikar F., Jaffar H.M., Khursheed T. (2021). Investigation of the anti-hyperglycemic and antioxidant effects of wheat bread supplemented with onion peel extract and onion powder in diabetic rats. J. Diabetes Metab. Disord..

[60] Kim J.S., Kim H.J., Lee E.B., Choi J.H., Jung J., Jang H.H., Park S.Y., Ha K.C., Park Y.K., Joo J.C. (2023). Supplementary effects of *Allium hookeri* extract on glucose tolerance in prediabetic subjects and C57BL/KsJ-*db*/*db* mice. Pharmaceuticals.

